# A Case of Occult Pistachio-Shell Endobronchial Foreign Body Causing Recurrent Pneumonia

**DOI:** 10.7759/cureus.37686

**Published:** 2023-04-17

**Authors:** Olawale Babalola, Venkatkiran Kanchustambham

**Affiliations:** 1 Pulmonology, University of North Dakota School of Medicine and Health Sciences, Fargo, USA; 2 Pulmonary Critical Care, University of North Dakota School of Medicine and Health Sciences, Fargo, USA

**Keywords:** pulmonology and critical care, persistent pneumonia, airway foreign body, endo bronchi, rigid and fiber-optic bronchoscopy

## Abstract

Endobronchial foreign body aspiration is a rare occurrence in adults, with a higher incidence in children. However, the possibility of such foreign body aspiration should not be overlooked in adult patients presenting with recurrent pneumonia symptoms, particularly when antibiotic treatment is ineffective. The diagnosis of occult endobronchial foreign body aspiration is challenging and requires a high degree of clinical suspicion, as it may not be associated with a history of aspiration. In this report, we present a case of recurrent pneumonia persisting for over two years, which was eventually diagnosed as an endobronchial foreign body due to occult pistachio shell aspiration. The foreign body was successfully removed through bronchoscopy. The diagnostic workup for recurrent pneumonia, including imaging and bronchoscopy, and the therapeutic management of endobronchial foreign body aspiration are discussed in detail. This case highlights the importance of considering endobronchial foreign body aspiration as a potential diagnosis in adult patients presenting with recurrent pneumonia, even in the absence of a history of aspiration. Early recognition and prompt intervention can prevent potential complications, including bronchiectasis, atelectasis, and respiratory failure.

## Introduction

An endobronchial foreign body is an uncommon diagnosis in adult patients compared to younger individuals. This is because a healthy adult has the neuromuscular competency to adequately manipulate the airway-esophagus transit, thus avoiding food particle aspiration, compared to the pediatric population [1]. However, there are instances where this mechanism might fail due to various pathologic and situational reasons, leading to the movement of particles, especially food into the respiratory tract. The anatomy of the right mainstem bronchi makes it more predisposed to the lodging of a foreign body [1,2]. The right main bronchus is shorter and wider thus making it an easy passageway for particles to lodge in the right lungs, especially the right lower lobe. Symptoms of endobronchial foreign body can be subtle enough to be misdiagnosed as a mild case of bronchitis [3]. Therefore, a thorough history and physical examination with appropriate diagnostic testing are important during the evaluation of patients. Ample time should be spent on history to uncover any episodes of aspiration as a brief resolution of post-aspiration symptoms might make patients omit important details.

## Case presentation

**Figure 1:**
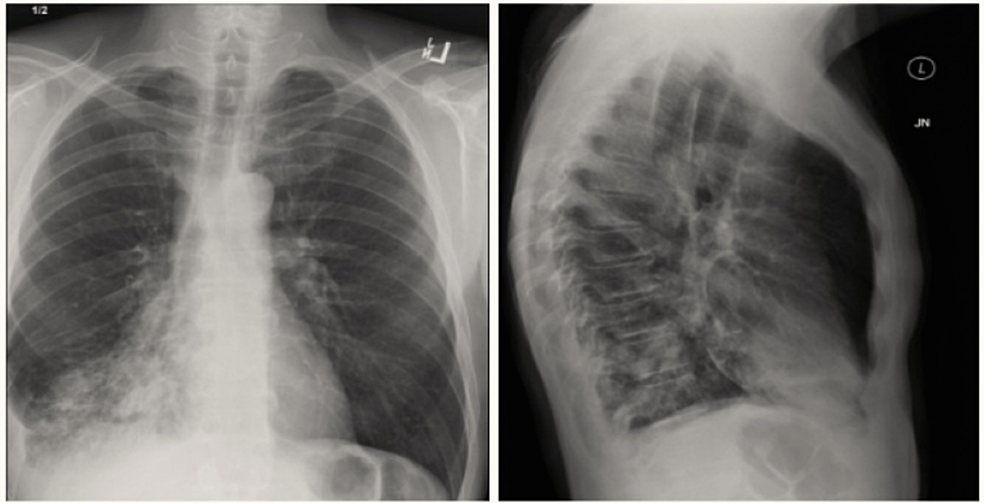
Chest X-ray showing right lower lobe consolidation

We present a case of a 57-year-old Caucasian male with 30-year smoking history who had presented to the family medicine clinic with a complaint of mild persistent cough. During the initial visit, an X-ray evaluation revealed right lower lobe pneumonia (Figure 1). The patient was sent home on azithromycin and a cough suppressant (codeine-guaifenesin) with instructions to call the clinic if his condition deteriorated. 

**Figure 2:**
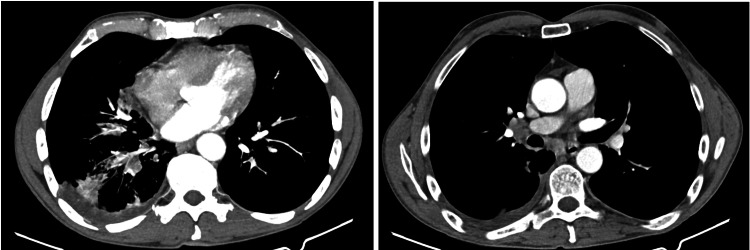
CT scan showing tree-in-bud pattern with consolidation (left) and hilar lymphadenopathy (right)

He again presented to the walk-in clinic three weeks later with a worsening productive cough, sinusitis, and nocturnal fever and chills. Computed tomography (CT) of the chest was performed which showed frank consolidation along the medial aspect of the middle lobe with surrounding patchy areas of tree-in-bud and reticular nodular opacities. There were also prominent right hilar, subcarinal, and right paratracheal lymph nodes (Figure 2). He was treated with two injections of intramuscular ceftriaxone followed by oral cefdinir. Follow-up was scheduled at six weeks during which the patient reported feeling better but with a lingering cough. He was told to return if symptoms worsens. Within the next six months of continuous waxing and waning cough, he developed an inguinal hernia which necessitated surgical intervention.

Eight months after the initial visit, he presented to the clinic with complaints of wheezing, productive cough, and occasional shortness of breath. No imaging was performed and a clinical diagnosis of bronchitis was given. He was sent home on a 10-day course of doxycycline.

**Figure 3:**
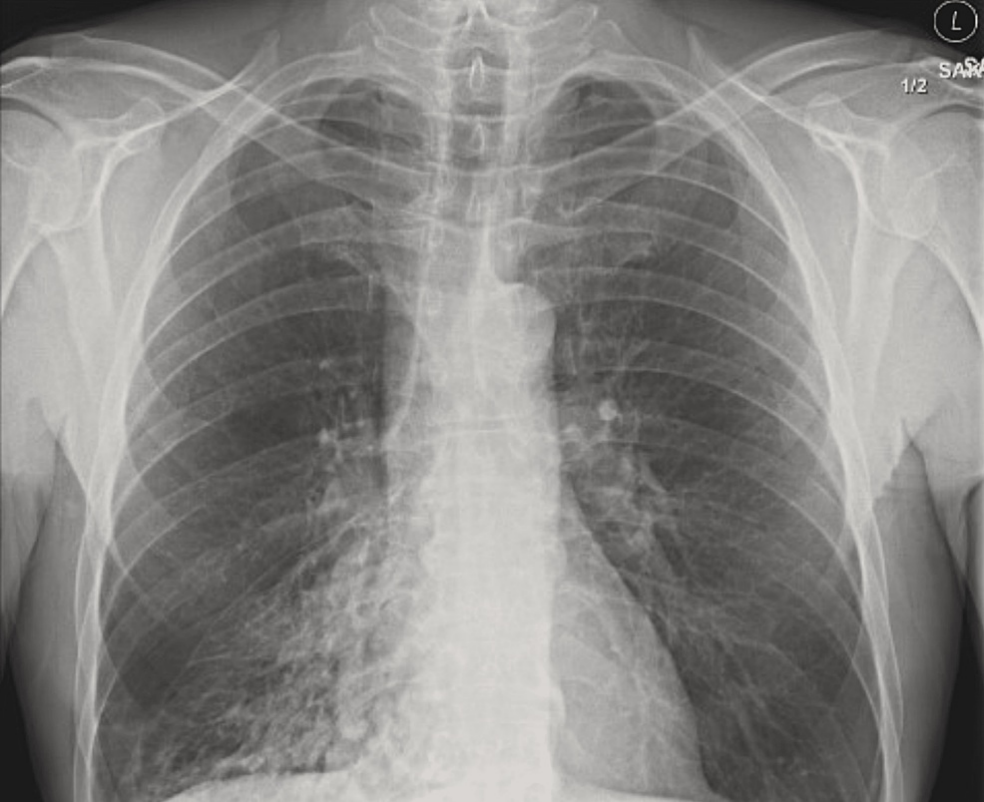
Chest X-ray showing patchy areas of consolidation on medial right lower lobe

Eighteen months after the initial visit, he presented with a worsening cough, fever and pleuritic chest pain. Chest CT and COVID testing were offered which the patient declined. Chest X-ray showed patchy consolidation in the medial right lower lobe (Figure 3). He was given a dual diagnosis of pneumonia and bronchitis and treated with IM ceftriaxone and oral doxycycline.

**Figure 4:**
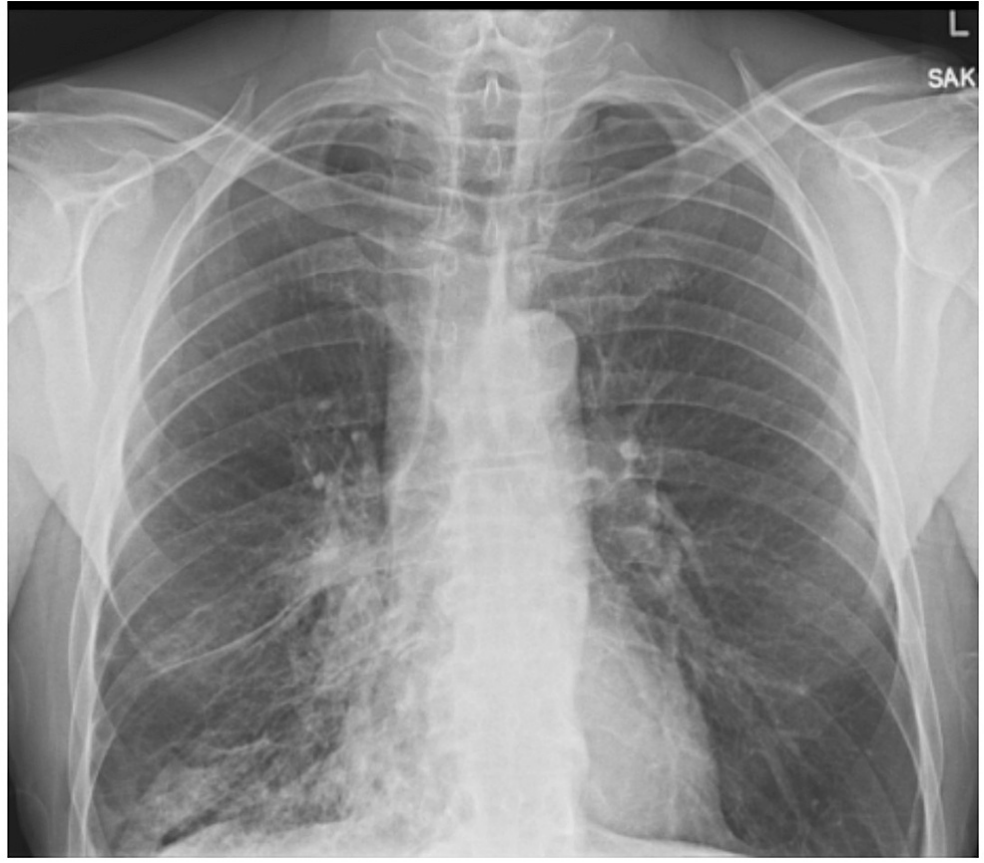
Right lower lobe pneumonia on X-ray

**Figure 5:**
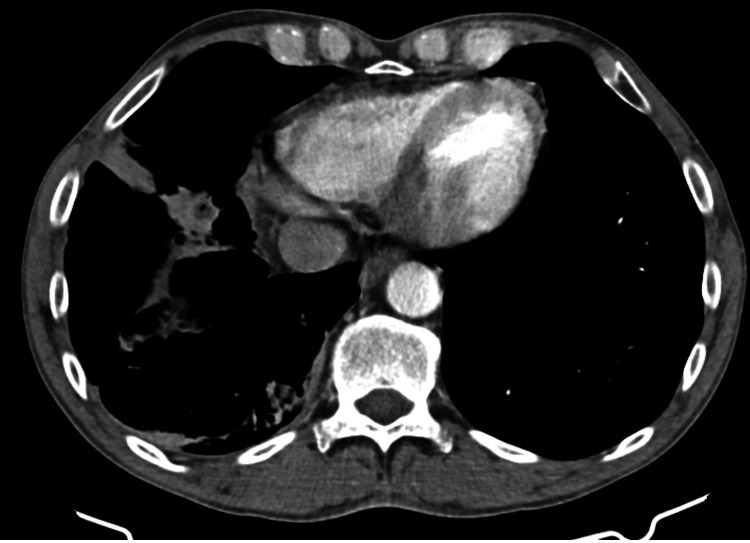
CT showing curvilinear hyper-density in the right lower lobe region

On month 23, he presented to the clinic with wheezing, shortness of breath and a harsh productive cough. Testing for COVID SARS2 virus was done which returned negative and an X-ray showed pulmonary consolidation in the right lower lobe with mildly hyperinflated lungs (Figure 4). Biochemical testing including complete blood count and basic metabolic panel results were within normal range. Patient was offered the option of hospitalization for further evaluation of recurrent pneumonia which he declined. Chest CT was performed which showed areas of nodular consolidation in all three lobes of the right lung with an enlarged mediastinal and hilar lymph node. At this point, a consultation was placed with pulmonology and bronchoscopy; tissue sampling was recommended as there was a high concern for malignancy especially in the setting of the patient's smoking history. Also, the presence of endobronchial foreign bodies was high on differentials. His CT was further reviewed at the pulmonology clinic and areas of curvilinear hyperdensity within the right lower lobe bronchus concerning for a foreign body were noticed (Figure 5).* *

Treatment

**Figure 6:**
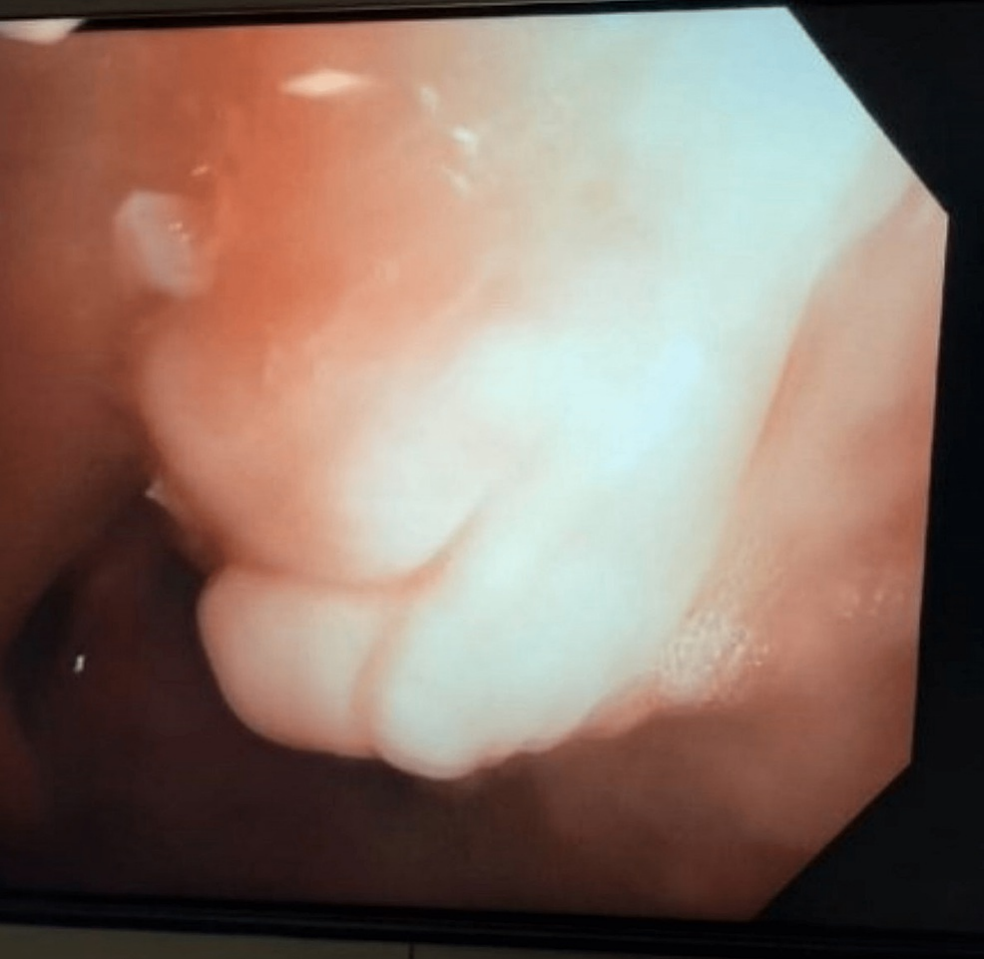
Endobronchial lesion obstructing the bronchus intermedius

**Figure 7:**
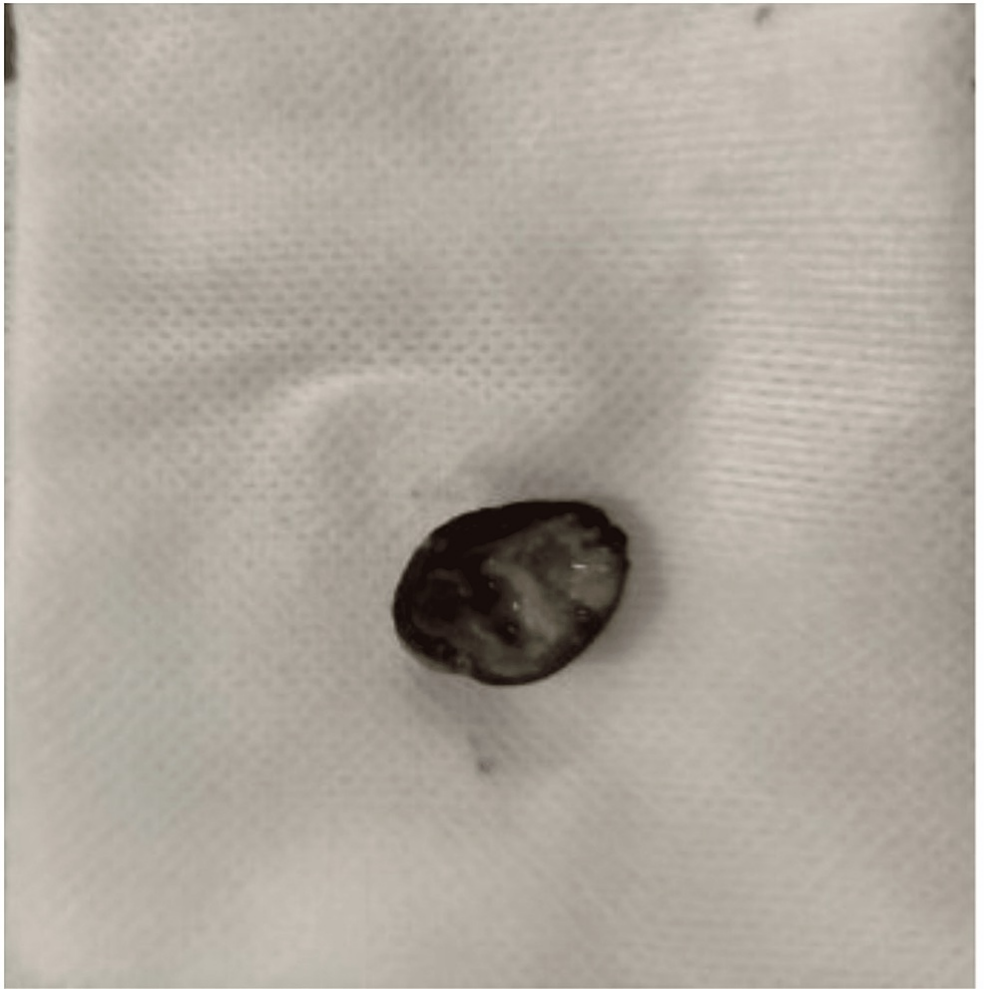
Foreign body (pistachio shell) removed during bronchoscopy

**Figure 8:**
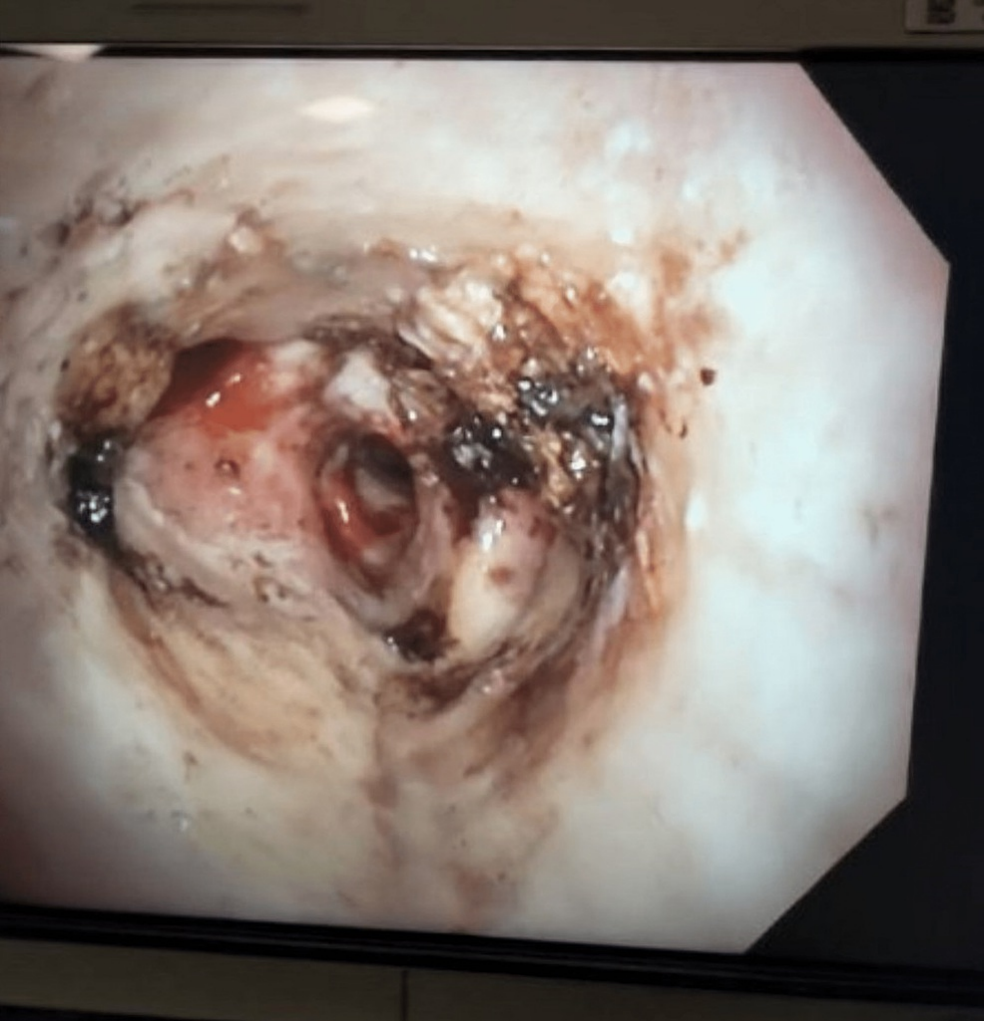
Bronchus intermedius after suction removal of foreign body

Bronchoscopy with cryotherapy - there was an endobronchial lesion obstructing the bronchus intermedius extending into the right middle and lower lobe (Figure 6). There was a significant amount of thick mucopurulent secretions surrounding the endobronchial lesion, this was suctioned clear. Using the cryoprobe, the endobronchial lesion appeared to be a foreign body - the shell of a pistachio was grabbed with forceps and removed (Figure 7). Inspection of the bronchus intermedius showed mild granulation tissue in the right middle lobe and right lower lobe superior subsegment opening, there was no bronchial stenosis or bronchomalacia from the foreign body (Figure 8). Cauterization and removal of granulation tissue were performed using cryoprobe and Argon plasma coagulation. Bronchoalveolar lavage (BAL) samples were obtained from the right lung and sent for culture. Enlarged lymph nodes were sampled via multiple fine-needle aspiration (FNA) biopsies using Cook 22 needle (Cook Medical, Bloomington, IN). The biopsied lymph nodes showed no malignancy and BAL cultures yielded no growth of bacteria, fungi, Nocardia or mycobacteria. Transbronchial tissue exam was negative for carcinoma.

Follow-up

A repeat bronchoscopy was scheduled six weeks later to rule out bronchial stenosis and for the removal of potential residual granulation tissue. Small granulation tissue immediately above the superior subsegment of the right lower lobe and before the right middle lobe opening were removed using cryotherapy and Argon plasma coagulation. Repeat transbronchial tissue sent for examination revealed residual granulation tissue and squamous metaplasia with focal atypia which was favored to be reactive. Patient was advised to follow-up in three months for a repeat CT-chest to monitor resolution. Unfortunately, the patient did not return for a follow-up visit. 

## Discussion

The incidence of endobronchial foreign body in adults is relatively low compared to children, thus the diagnosis is often overlooked by clinicians on initial contact. In a single-center study, the mean age of patients suspected to have foreign body aspiration was 8.1 ± 14.0. In this study, 88.3% of cases were suspected in pediatric patients compared to 11.7% in adults [1]. The lack of commonality of this condition in adults may be responsible for its omission in the differential diagnosis of adult patients presenting with airway symptoms. Medical history is the most crucial factor in the diagnosis of foreign body aspiration [1]. Clinicians often assume adult patients should be able to avoid or, at least, self-report aspiration. With such an assumption, direct questioning of aspiration episodes is often skipped during history taking. Perhaps, some patients might feel some degree of embarrassment about the self-reporting aspiration of food content. Therefore, clinicians need to perform a thorough history, especially in patients presenting with recurrent respiratory symptoms. In instances where the aspiration episode remains unconfirmed after a thorough history, a high degree of suspicion for an occult foreign body process should be maintained.

Airway aspiration has a bimodal distribution of larger incidence in children 1-2 years and adults older than 70. Common predisposing factors in adults include dysphagia, movement disorder, alcohol intoxication, or poor dentition [2]. While foreign body aspiration is a medical emergency in children less than 3 years of age, the presentation of foreign body aspiration in adults is often subtle without pathognomonic features. Cough is the most common symptom with initial episodes of irritative, spasmodic attacks which are slightly suppressed after a foreign body lodges permanently in the endobronchial lining. In fact, the patient might be completely asymptomatic upon presenting to the emergency department as the foreign body might have progressed distally. The resulting sequel is recurrent pneumonia, bronchiectasis, atelectasis, and pneumothorax which might be associated with mortality in later periods in adult patients [1,3,4]. Therefore, a history of cough that starts during eating accompanied by breathing difficulty and cyanosis should raise a high suspicion for foreign body aspiration [1]. 

Imaging studies used in evaluating foreign body aspiration are mainly chest X-ray, thoracic CT and bronchoscopy. Radiopaque bodies can easily be seen on X-ray; however, most aspirated foreign bodies are non-opaque [1,5,6]. Therefore, the use of chest X-rays is limited as a diagnostic tool in evaluating foreign body aspiration. Bronchoscopy remains the leading therapeutic option for the management of foreign body aspiration. Rigid bronchoscopy (RB) or flexible bronchoscopy (FB) can be employed in the removal of foreign bodies, although the success rate in RB (99%) is higher than in FB (86%-91%) [7]. However, recent studies have demonstrated the value of FB as the first approach with the employment of RB only in difficult cases [8]. It is important to note that rigid bronchoscopy allows for easy manipulation of proximal airways, hence, it is the instrument of choice for central airway bronchoscopy [2]. Most importantly, the selection of the technique to use is dependent on the available equipment and expertise of the clinician performing the procedure [7]. 

Several complications can occur during foreign body removal. Central airway obstruction, bronchospasm and migration of foreign body fragments are important to watch closely as these can lead to rapid deterioration of the patient's respiratory status [2,7].

## Conclusions

A prolonged delay in diagnosing an endobronchial foreign body increases the risk of developing complications in addition to the increased cost incurred due to multiple visits to the healthcare facility. Therefore, an early aggressive approach is encouraged in terms of imaging and invasive intervention in patients with recurrent pneumonia with a high suspicion of an endobronchial foreign body. To reach such a level of clinical suspicion, it is imperative to conduct history in a thorough and non-judgmental pattern. Also, fibre-optic bronchoscopy remains the gold standard diagnostic tool for foreign body aspiration in adults, therefore, referral to pulmonology should be considered early in the management of patients with recurrent pneumonia. 
